# Non-Invasive Physiological Indicators of Heat Stress in Cattle

**DOI:** 10.3390/ani11010071

**Published:** 2021-01-02

**Authors:** Musadiq Idris, Jashim Uddin, Megan Sullivan, David M. McNeill, Clive J. C. Phillips

**Affiliations:** 1 Department of Physiology, Faculty of Veterinary and Animal Sciences, The Islamia University of Bahawalpur, Punjab 63100, Pakistan; 2 Centre for Animal Welfare and Ethics, School of Veterinary Science, Gatton Campus, The University of Queensland, Gatton, QLD 4343, Australia; j.uddin@uq.edu.au (J.U.); d.mcneill@uq.edu.au (D.M.M.); 3 School of Agriculture and Food Sciences, The University of Queensland, Gatton, QLD 4343, Australia; Megan.Sullivan12@outlook.com; 4 Curtin University Sustainable Policy Institute, Kent St., Bentley, Perth, WA 6102, Australia; clive.phillips@curtin.edu.au

**Keywords:** body temperature, heat stress, hyperthermia, faecal corticosteroid, infrared thermography

## Abstract

**Simple Summary:**

Heat stress is a common cause of poor welfare in cattle and is expected to increase with climate change. The biggest challenges are to feedlot and dairy cattle in hot climates but determining whether cattle are heat stressed can be difficult due to the invasive and time-consuming nature of established methods of measurement, such as rectal temperature, and the lack of specificity of some measures, such as cortisol in plasma or faeces. We review the various methods of non-invasively measuring heat stress in cattle and highlight the value of infrared thermographic imaging for the accurate and rapid determination of external body surface temperatures. Further research options to establish thresholds and optimum methodology for these non-invasive measures and their use in different cattle types are identified. Their use promises to accelerate response times to cattle experiencing heat stress under intensive management conditions.

**Abstract:**

Cattle are susceptible to heat stress, especially those kept on high levels of nutrition for the purpose of maximising growth rates, which leads to a significant heat increment in their bodies. Consequences include compromised health and productivity and mortalities during extreme events, as well as serious economic loss. Some measures of heat stress, such as plasma cortisol and temperature in the rectum, vagina, or rumen, are invasive and therefore unlikely to be used on farms. These may cause additional stress to the animal due to handling, and that stress in itself can confound the measure. Consequently, it is desirable to find non-invasive alternatives. Panting score (PS), cortisol metabolites in faeces, milk, or hair, and the infrared temperature of external body surfaces are all potentially useful. Respiratory indicators are difficult and time consuming to record accurately, and cortisol metabolites are expensive and technically difficult to analyse. Infrared temperature appears to offer the best solution but requires further research to determine the thresholds that define when corrective actions are required to ensure optimal health and productivity. Research in this area has the potential to ultimately improve the welfare and profitability of cattle farming.

## 1. Introduction

Welfare and productivity of cattle can be severely compromised in hot environments, especially for those kept in subtropical and tropical regions where animals are regularly exposed to high ambient temperature and/or relative humidity for prolonged periods [1]. In the literature, either “heat load” or “heat stress” are used to describe the animal’s suffering under hot conditions [2,3]. Cattle exposed to prolonged heat stress reduce their feed intake, milk production [4,5,6], growth and welfare [7], reproductive performance [8], health, and immunity. Consequences include serious economic loss and mortalities during extreme events [9,10]. Almost 20 years ago heat stress in the US beef industry heat stress resulted in more than 300 million dollars of loss to the national economy [11]. In Australia, a 16.5-million-dollar loss to feedlot industry has been estimated over the summer period of 2006 [12] and more recently during 2019–2020, high temperatures caused not only heat stress but also bushfires affecting the livestock industry, an exact estimate of the damage is awaited. Thermal acclimation to a heat stress event helps to maintain productivity but it is variable for different beef breeds, for example it has been reported as 9 and 14 days for Angus and Charolais, respectively [13].

The body temperature of animals increases in hot environmental conditions, including high humidity and/or high solar radiation, particularly when ambient temperature exceeds the upper critical temperature [14]. Animals suffering with these conditions attempt to cope by dissipating heat through different physiological means, that are coordinated by the hypothalamus [14,15]. These centre on heat dissipation from the surface of the skin through increased peripheral circulation, vasodilatation, and sweating [16].

The endocrine response to heat stress is mainly reflected in elevated glucocorticoids (cortisol), aldosterone, antidiuretic hormone, thyroxine, prolactin, and growth hormone (GH) [17,18]. The benefits of using faeces that has been discharged from the body as a suitable biological material for estimation of cortisol metabolites is that it provides a longer-term non-invasive approach, compared with conventional blood sampling, which is a short-term assessment and an invasive procedure, requiring handling of animals [19,20,21]. Higher plasma cortisol concentrations in cows were reported in earlier studies during acute heat stress conditions [22,23], but increased concentrations of cortisol metabolites in faeces have also been shown in animals exposed to acute heat stress conditions [18].

Cattle core body temperature ranges from 38.0 to 39.3 °C during thermoneutral conditions [16,24]. Different methods are used for estimating core body temperature, by approaching through the rectum [24,25], vagina [26,27,28], tympanic membrane of the ear [29], rumen [3,30], or using infrared thermography of external body surfaces [31,32]. These all differ in their speed of response to heat stress, accuracy, and degree of invasiveness [33], and are discussed individually in this review.

Climate change means that cattle will be increasingly exposed to heat stress [34]. Economic losses, resulting from poor production performance of heat stressed animals, could be reduced by predicting the exact time for the implementation of corrective actions to manage the risk of heat stress. This could be accomplished through better understanding of the cattle responses during heat stress conditions. The measurement of faecal and hair cortisol hormone metabolites and infrared thermography are among the most recent approaches, with the added advantage that they are non-invasive. We hypothesized that heat stress detection in cattle is supported by a growing literature that will eventually enable farmers to control this source of stress. This review presents the current state of knowledge, using an informed collection of results obtained from recent research to highlight the possibilities for using non-invasive approaches to detect heat stress in cattle.

## 2. Stress Response to Hot Environment

Selye [35,36] demonstrated a common stress response, driven by the adrenal gland, to different stressors and defined stress as the biological responses upon exposure to an adverse environmental condition. The term “stressors” was used to define adverse environmental conditions. Stress does not always result in overall detrimental effects for animals [37], as this could be an indication for adaptation to a new environment [35].

Extreme environmental circumstances, hot or cold, are potential stressors [35]. In particular, high temperatures often lead to low productivity, lowered immune responses, high morbidity, and mortality. The exposure of animals with hot environmental conditions are described using one of the two terms, either “heat load” or “heat stress” [2,38]. Heat load would appear to be an indication of the levels of factors that are supplying the environmental impact on the animal and the heat stress is how the animal responds.

Heat stress affects cattle bioenergetics [17,39,40], and particularly afflicts rapidly growing beef cattle and high lactating cows because of their requirement for a high plane of nutrition and the significant heat increment arising from rumen fermentation [1]. Cattle respond to heat stress through increased heat dissipation [17] by physiological and behavioural thermoregulatory mechanisms [16,38,39]. These mechanisms involve increased heat dissipation through sweating and peripheral circulation, increased respiration, panting, and also reducing feed intake to lower metabolic heat [16].

## 3. Thermoregulatory Mechanism

**Figure 1:**
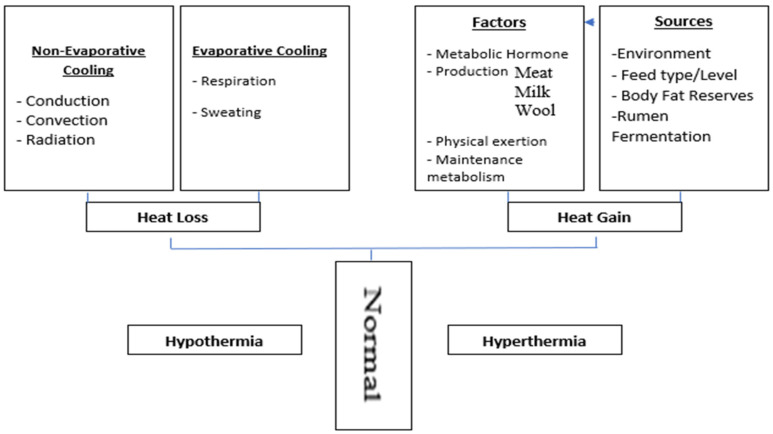
Thermoregulatory mechanisms adapted from Tait [41] and Yousef [42].

In homeotherms, initial thermoregulatory response is to maintain and restore constant core body temperature, allowing heat to dissipate from the body through insensible and sensible thermal heat exchange. Insensible heat exchange, also known as evaporative heat loss, uses evaporation, that involves a vapour gradient for thermal heat exchange. Sensible thermal heat exchange also known as non-evaporative heat loss that involves heat loss through a thermal gradient. Sensible thermal heat exchange includes convection, conduction, and radiation [7]. Thermoregulatory mechanisms have already been reviewed in literature [7,41], however, for a quick review, these mechanisms are detailed here in Figure 1.

## 4. Environmental Factors Related to Heat Stress in Cattle

**Figure 2:**
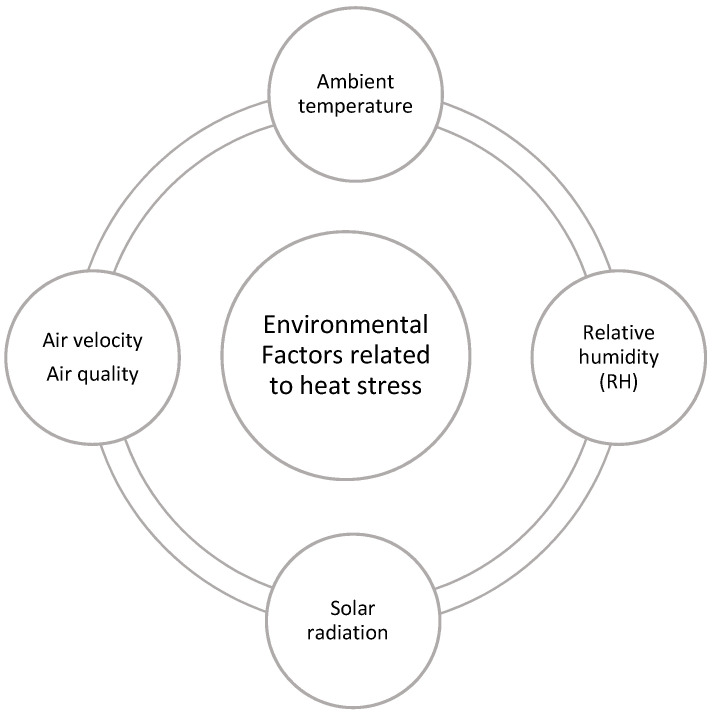
Environmental factors related to heat stress in cattle.

Environmental factors affecting cattle welfare due to heat stress include ambient temperature, relative humidity, and solar radiation, having direct or indirect impact on cattle welfare. Although brief exposure to hot environmental conditions may result in an insignificant impact on production performance, during sustained exposure to hot conditions welfare problems can arise, especially for the high-producing dairy and feedlot cattle [17,43]. The animal’s climatic environment is complex, especially in outdoor conditions. The impact of environmental factors related to heat stress in cattle has already been reviewed in literature [44], however for a quick review, these factors are outlined in Figure 2.

## 5. Cattle Responses to Heat Stress and Their Measurement

Cattle respond to heat stress through adapting various behavioural and physiological responses. The cattle responses to any change in the climate and/or environment are referred to as the process of acclimation and adaptation, respectively [24]. Acclimation and adaptation to hot climatic conditions involve phenotypic changes that are critical for sustainable livestock production and may take several generations [2,3,45]. Hot climatic conditions induce gene expression changes and endocrine and metabolic adaptive responses to improve thermo-tolerance [2,16] but can be accompanied by reduced production potential [46].

The process of acclimatization, a coordinated response to several individual thermal stressors simultaneously such as temperature and humidity, begins within three to four days after the exposure to hot conditions [47,48,49]. In an acute heat stress event of one or two days, cattle adapt short-term adaptive ways such as drinking more water, increased respiration, and sweating to dissipate heat from the body. However, in chronic heat stress events, as short-term adaptive ways fail, body temperature gradually increases, resulting in reduced feed intake and poor production [1,50]. Increased heart rate [51], sweating rate [52], standing or lying times, and pattern of feeding [3,38,39,53] can all indicate chronic heat stress.

### 5.1. Respiration Rates

Sweating and panting are the major routes for evaporative cooling in cattle, particularly sweating [54]. Respiration rate and PS serve as early indicators of increasing heat stress [1] and provide an easy method for the non-invasive and distant assessment of heat stress response [7], unless cattle are more than about 30 m away [55]. Respiratory changes can comprise 30% of total heat dissipation and have minimum lag time of 1–2 h [24,54,56]. Increased respiration rate by approximately 4.3 breath per minute above baseline respiration rate i.e., 60 breaths per minute has been reported by Hahn et al. [48] with each degree °C rise above the threshold temperature of 21.3 °C.

This increase in respiration rate in cattle may go up to 200 breaths per minute during heat stress events [44], reflecting an imbalance between heat accumulated and dissipated [57,58].

A panting score serves as a useful non-invasive observation tool to assess cattle responses to hot environmental condition as an alternate tool to other standard assessment methods [39,59]. The panting score can be helpful to assess an individual, or a group of animals’ responses related to heat stress if a mean panting score is calculated for these animals [55,58,60]. However, being a qualitative method with only a small number of scores, it can be difficult to attribute scores accurately, especially if researchers or farmers are not trained. There is also likely to be considerable variation between individuals in the use of panting at different levels to dissipate heat.

### 5.2. Body Condition and Growth

The feedlot industry relies on body condition and growth as an indirect way for judging production performance and welfare of feedlot cattle, including during heat stress [61]. However, its ability to determine welfare is confounded because cattle with high body condition score are more susceptible to heat stress [62], increasing their respiratory and panting rates more [57].

Heat-stressed cattle spent most of their body energy to maintain homeostasis, reducing energy reserves available for growth [63,64] and increasing the required days in feedlots to achieve market specification targets [65].

### 5.3. Health Status

Heat stress increases the risk of pneumonia and internal organ damage, especially in feedlot cattle [66]. Animals with compromised health are also more susceptible to heat stress [67]. For example, feedlot cattle with a previous history of respiratory pneumonia experience on average a 10% higher heat stress [57]. Mortality is also increased in heat stress. In the United States, more than 4000 cases of feedlot cattle mortality were reported in Iowa state following a severe heat stress event during 2006 [66]. High mortality rates may be an indirect result of a depressed immune system and reduced resistance to pathogens. Delays in circulatory and skin surface lymphocyte production following heat wave events could explain increased illness [68]. However, as with other measures, the accurate measurement of a relevant parameter is dependent on having a scoring system that has been comprehensively validated. Health records are often bimodal (yes/no) in nature and not easily analysed unless there are large numbers of animals.

### 5.4. Endocrinological Changes

The endocrinological changes play a crucial role in the metabolic response to heat stress through neural and glandular secretion of hormones [69], in particular glucocorticoids, antidiuretic hormone, growth hormone, thyroxine, prolactin, and aldosterone [70]. In acutely heat-stressed cattle, concentrations of antidiuretic hormone [71], prolactin [72], glucocorticoids and catecholamine increase [73], and aldosterone decrease [23,74]. However, in chronic heat stress, glucocorticoids, growth hormone, and thyroxine may remain the same or decrease [18,75]. The shift in hormone concentrations during acute heat stress event is associated with decreased concentration of electrolyte and water balance, as water and electrolyte losses occur during panting and sweating [74,76]. Higher concentrations of prolactin have been reported to play a vital role in the sodium and potassium balance during hot environmental temperatures [23,74]. The differential response in glucocorticoid production may be because in chronic heat stress elevated levels of progesterone and reduced glucocorticoid production may reflect reduced conversion of progesterone to glucocorticoids [77]. However, during a chronic heat stress event, higher concentrations of catecholamine have also been reported, perhaps due to increased activity of sweat glands, that are regulated through sympathetic innervations in cattle [78,79,80]. In heat-stressed cattle, decreased aldosterone levels have been observed during both acute and chronic heat events, probably due to the excretion of more sodium via urine, following increased consumption of water.

#### Cortisol Metabolites

Blood may be sampled for analysing endocrine changes, but that in itself is a stressful and an invasive procedure. The estimation of milk cortisol can be used as an efficient non-invasive method to assess stress response in lactating cows. The concentration of cortisol metabolites in milk collected at the same time as blood samples correlates well with plasma cortisol [81].

The measurement of cortisol in faecal samples is advocated by Palme et al. [19] and Palme [21], and the use of hair by Heimbürge et al. [82] and Mastromonaco et al. [83]. Faecal cortisol metabolites provide a relatively acute assessment of stress, with a time lag of 8–16 h [84]. However, hair cortisol is a biomarker of chronic stress, which may be from weeks to months depending upon length of hair and growth rate [82]. Increased faecal cortisol metabolites were observed in cattle exposed to acute heat conditions [18]. Hence in studies that involve estimation of cortisol metabolites to assess stress responses in cattle faeces acute heat stress should be considered as one of the confounding factors [18]. Veissier et al. [15] reported increased faecal and milk cortisol metabolites as the heat load index increased from 50 to 79. The milk cortisol concentration in dairy cows without shade is higher in the evening than in the morning, when the heat load index is above 80. Similarly, an increased concentration of faecal cortisol metabolite, correlated with increased respiration rate, has been reported in feedlot cattle when they were exposed to hot environmental conditions (daily max. T_a_ 35 °C and Relative Humidity 50%; Temperature Humidity Index 77), when compared with a thermoneutral period under environmentally controlled experimental conditions [85]. In a chronic heat stress study, Nejad et al. [86] reported that hair cortisol metabolites increased from 6.3 to 24.2 pg/mg in dairy Holstein cows when ambient temperature increased from the coolest (18 °C and 65% humidity) to the hottest conditions (38 °C and 91% humidity).

Although cortisol metabolites may be a useful experimental tool to assess cattle responses to heat stress, as a field tool they suffer from the complexities related to laboratory establishment at cattle facilities, sample numbers to be processed and estimated time needed to receive results after analyses from the existing commercial laboratories.

### 5.5. Body Temperature Changes

Core body temperature (38.6 °C, range 38.0–39.3) in cattle [16,24] indicates the temperature of the most important body organs: the heart, liver and brain. Indirect measures of core body temperatures include rectal [24,25], vaginal [26,27,28], tympanic [29], or rumen temperature [3,30]. All differ in their speed, accuracy, and invasiveness [33]. They should be measured while the animal is resting and has low productivity. Cattle as homoeothermic animals can maintain a stable core body temperature by modifying metabolic heat production and/or heat loss [87]. However, apart from heat stress, deviations in core body temperature from normal can derive from fear, pain, stress, anxiety, and disease [88,89,90,91,92,93]. Rectal or vaginal temperature is used as a conventional “gold standard” measure of core body temperature, recorded by placing a thermometer into the rectum or vagina for approximately 30 s. The two are highly correlated in dairy cows at least [94]. Recording requires displacement of cattle from their social group and restraint in a crush, which can affect their welfare [95]. Measurement of rectal temperature is influenced by the procedure adopted—the depth of penetration of the thermometer into the rectum and presence of faeces [96,97].

Thermal data loggers can be used to measure body temperature by attaching them with the animal’s skin surface by either plaster, glue, or any other fastening material. Other options include implantation of a logger’s electrode or its placement close to the tympanic membrane of the eardrum, or within a rumen bolus, metal harness or a vaginal insert, such as a controlled internal drug release device (CIDRs) [92,93,98]. The loggers record and store body temperature at pre-determined time intervals, and after removal the stored data are downloaded. Data loggers also have a telemetric option to transmit recorded data in real time to a user-defined receiver software for further calibration and/or processing by an algorithm. Conventional rectal or vaginal temperatures differ from body temperatures recorded by data loggers in other parts of the body. For example, the internal temperature of the rumen is approximately 1 °C higher than core body temperature, because ruminal micro-organisms produce heat by fermentation of digesta [99]. Conversely, the udder temperature is 1 °C lower than core body temperature. Whilst there are many techniques for the measurement of core body and or external body surface temperatures by thermal data loggers, a consistent limitation is that the majority of them require handling and restraining of the animal. Loggers need to be inserted and maintained, and all require invasive procedures for retrieval of data [100]. These confounding factors affect the relationship between measured temperatures and cattle stress [100,101,102]. Even with telemetry, there may be interference in the data transmission, and thus the signal of the sensor placed in/on the animal’s body must be often reinforced by an antenna, which may require an external power supply [103]. The amount of time that the animal needs to be restrained for inserting the data logger is minimal compared to the amount of time that they can be in place recording body temperature. The temperature data collected around the times of insertion and retrieval can be removed from analysis and use only the time when the animal is in a relatively normal or free behaving state. Additionally, using data loggers and or telemetry may be impractical at a commercial herd level for routine screening of body temperature. Such devices may be only useful in fundamental studies to investigate acute stress responses, such as during transportation or de-horning.

Surface body temperatures are usually measured in the sublingual, axilla, groin, neck, ear, thorax, and forehead regions [100,104]. A rise in ambient temperature results in increased peripheral circulation, sweating, and respiration rate to cope with the environment [24]. Surface temperature is usually lower than core body temperature, because the amount of blood circulation is progressively reduced from core to periphery, the size of blood vessels being comparatively smaller in the peripheral area, giving more opportunity for heat loss. Whilst thermal data loggers have been commonly used to monitor surface temperatures, infrared thermography is rapidly developing as a promising alternative [102,105].

#### Potential of Infrared Thermography to Measure Body Temperature

Infrared thermographic cameras measure body temperature of animals from proportional emissions of radiated heat from the external body surfaces. These derive from a combination of changes in core body temperature but also changes in the blood flow sub-surface. Heat emissions are detected by the camera’s infrared sensors and displayed as a thermogram of pixels varying in colours or shades that indicate different infrared temperature (IRT) [89,106]. Emissivity of objects varies from 0 to 1, since some absorb and emit no radiation, such as a mirror, and some absorb and emit all radiation, such as a black body. The emissivity of cattle external body surfaces ranges from 0.93 to 0.98, depending on skin and hair colour, and density of hair [107]. This method is non-invasive and has greater potential for automation than rectal or vaginal temperature [108,109,110]. This rapid and reliable non-invasive tool can screen large numbers of animals with minimal to no restraint, providing a more efficient use of labour resources [111].

Minimum, average, and maximum IRT of user-defined external body surfaces are determined for each thermogram using the relevant software. Changes in the maximum IRT of external body surfaces are better associated with changes in the behavioural responses of emotions, lactation variables, and metabolism than average or minimum IRT values [31,32,112,113,114,115]. Minimum and average IRT calculation are prone to variation in positioning of the frame of the thermogram, as well as external factors such as adhesion of water droplets and mud or faeces on external surfaces. The use of a single pixel to estimate a temperature, as is the case when maximum IRT values are used, is subject to pixel error [115], but this usually has lower variance than that of minimum or average [32].

Measuring the IRT of ears and muzzles is problematic due to frequent movement of ears and the presence of ear tags blocking emissions from the skin, and the muzzle region often has debris or excess moisture that could affect the reflectance spectra that the thermal camera detects. The maximum IRT of the eyes, particularly the skin around the inner corner of the eye socket, reflects core body temperature in cattle well [116], perhaps because the eyes are located close to the hypothalamic thermosensitive site, which reduces lag time in response. Additionally, IRT of eyes was not influenced by ambient temperature [116], and eyes have superficial capillaries beds in the *caruncula lacrimalis* and posterior border of the eyelids, which are highly innervated by the sympathetic nervous system [117,118,119]. Moreover, eyes have retinal blood vessels similar to the important brain vessels, and choroid vessels and ciliary processes, which are similar to the small intestine and kidney. The IRT of eyes and rectal and vaginal temperature are moderately correlated to each other: In multiparous, non-lactating, pregnant Senepol cattle correlation between IRT of eyes and rectal and vaginal temperature were 0.58 and 0.52, respectively [120,121]. IRT data of ocular and rectal temperatures are normally distributed, but in one study, the former was on average 2 °C lower than rectal temperature [116].

Given the importance to the eye IRT as a proxy of core body temperature, Uddin et al. [31] investigated the relationship between eye IRT and laterality of previously selected extremely lateralised (left or right side passing a person in the lane) lactating dairy cows. They reported a positive relationship between eye IRT and right laterality, and a negative relationship between eye IRT and milk fat content, which suggests that anxious cows had higher eye IRT and lower milk fat content. Previous studies indicated that cows who passed the person in the lane on their right side (right laterality) were more anxious than those passing on the left side, as evidenced by a higher crush score, flight speed, raised tail, and sniffing behaviours [122,123]. Moreover, Rogers [124] reported that the fight or flight response of an animal is mainly controlled by the right side of the brain. The right side of the brain is connected to the left eye and passing on the right allows an animal to view the potential threat more easily with their left eye [125]. Similarly, Lees et al. [126] identified that temperament of beef cattle influences the regulation of body temperature, while Lee et al. [93] reported that pharmacologically induced anxious beef cattle had higher rectal temperature than calm cattle. Therefore, changes in the IRT of eyes and rectal temperature may be an indication of similar physiological responses to stress.

However, in another study on randomly selected lactating dairy cows [127], the relationship between laterality and body temperatures, measured by IRT and rectal temperature, was not observed. Rather, both temperature measures were related to flight speed behaviours, but in an opposite direction. IRT of eyes was associated with flight speed in a negative correlation, but rectal temperature and flight speed was associated in a positive correlation. The IRT, laterality, flight speed, and rectal temperature in the Uddin [127] study were recorded on different days, not simultaneously. In contrast, the Gloster [116] study, which claimed that eye IRT was a proxy of core body temperature, measured IRT and rectal temperature simultaneously. Another discrepancy in the methodology of these two studies was that Uddin et al. [127] investigated the relationship of body temperature with behavioural responses in the presence of threatening stimulus, which was not the case in Gloster’s study [116], who studied the relationship of IRT, rectal temperature of cattle in a normal environmental condition. Therefore, our understanding is that IRT of cow external body surfaces always indicates surface temperature, which is linked to peripheral temperature more than core body temperature, particularly in the presence of environmental or emotional stress. Our recommendation for future research is to collect IRT and RT data simultaneously.

IRT data are more reliable than rectal temperature in the measurement of a cow’s body temperature, as suggested by Uddin [127], who investigated the repeatability of IRT and rectal temperature, recorded in three periods, one month apart in each period over three consecutive months. They reported that the eye IRT measure was strongly repeatable between periods, but rectal temperature was not.

The IRT of limbs appears to reflect peripheral temperature more than eye IRT. Peripheral temperature measurements confirmed greater variability as they form a buffer between the core body and the ambient temperature, mainly through convection [87,128]. Biologically, the IRT of each external body surface region varies according to local tissue metabolism and conduction, blood flow, and the ability of an object to absorb and emit radiation [129]. Limbs are important sources of heat loss in cattle [130,131]. Changes in peripheral IRT are more evident at the coronary band than other body parts, because it is a muscular structure actively involved in the movement of the animal and supporting the weight of the body, with profuse blood circulation to provide nutrition to the hooves [88,116,132,133]. Uddin et al. [31,127] identified consistent negative relationship between limb IRT and waiting time in two different studies, confirming that relaxed cows had lower external body surface temperature in the coronary band of forelimb. Thus, IRT of key external body surfaces shows potential to estimate changes in the body temperature of animals in response to changes in the environment, and IRT of eyes has positive correlations with commonly used rectal and or vaginal temperature.

The IRT measurement of the effects of the environment on external body surfaces is potentially confounded with emotional state. For example, the muzzle IRT in cattle decreases during positive emotional arousal, particularly when an animal handler gently strokes the animal [134]. The IRT of the eye region could be even more responsive to emotions than the muzzle. That may be a consequence of the eye being much closer to the emotional processing centres in the brain hemispheres and the small area around the medial posterior palpebral border of the lower eyelid and the *lacrimal caruncle,* both of which have rich capillary beds innervated by the sympathetic system. This enables them to actively respond with increased temperature to changes in blood flow during threatening situations. The IRT of the eye is easily measured without the interference of hair and is a more consistent measure of IRT changes than the muzzle, ear, body, and hooves in response to negative emotional stimuli and early signs of disease in cattle [135]. There is also evidence that the left eye of cattle responds more to sunlight than the right eye [91]. However, not all types of exogenous hypothalamic-pituitary-adrenal axis (HPA) stimulation increase the maximum IRT of the left eye similarly [116]. In response to two catheterisations, Stewart et al. [117] reported greater IRT responses the second time, suggesting that the cows had anticipated the stress. IRT measures of the feet of cattle also have potential to estimate feed efficiency, due to the correlation with heat loss [128]. Inefficient cows tend to have higher IRT, for example on their feet [117] or teats [128]. These measures have potential to measure the effects of heat stress on efficiency of cattle production.

## 6. Confounding Factors Associated with Infrared Thermography

Successful determination of IRT of external body surfaces depends on effective minimisation of confounding factors, e.g., those related to skin and hair colour, emissivity of the skin, unexpected movement, health status, and time of feeding, and external factors such as ambient temperature, relative humidity, sunlight, wind speed, distance between camera lens and measuring objects, and angle of camera positioning [89,90,91].

There should be at least a 10-min acclimatization period before capturing the thermogram [136], because peripheral blood circulation increases during the movement of animals. Hence, body temperature changes could produce thermograms with artificially high IRT [137]. Skin and hair colour and density of hair also have an effect on IRT measurements. Hair acts as an insulator and decreases the emission of infrared radiation [137,138]. Although animals with slick hair have been reported to have superior thermoregulatory capabilities probably due to increased sweating [139], slick hair can still have an effect on IRT measurements. For this reason, IRT at a hairless skin area such as the muzzle or eye usually gives a higher range of IRT than a hairy one [140]. Cattle with black hair absorb and emit more solar radiation compared to those with white hair, because black hair has a higher emissivity in visible wavelengths and consequent higher IRT in captured thermograms [91,141]. Church et al. [91] reported a dramatic effect of hair colour on the IRT measurement during solar loading—white parts of the head were 35 °C, while the black parts were 50 °C. The presence of any focal lesions in the external body surfaces and recent injections into the muscle could also influence IRT measurements [140], as inflammation of underlying tissues alters the amount of radiated heat by changing metabolism and circulation in these tissues [142].

Age and physical status of animals should also be considered as an influential factor for the IRT measurements. Lactating cows have higher body temperature than dry cows, and early lactating cows have comparatively higher body temperature than late lactating cows. This is because young and or early lactating cows have higher tissue metabolism and growth rate and higher metabolic heat production than late lactation cows [143,144]. Oestrus cows have increased physical activity and vaginal blood flow, an luteinizing hormone (LH) surge and ovulation, and secretion of progesterone in the luteal phase, all of which contribute to the enhancement of body temperature [145,146]. A circadian rhythm, which indicates a physiological fluctuation of core body temperature of animals, occurs at regular intervals (approximately 1.5 h) [99,128]. Lowest core body temperatures also occur in the early morning and reach maximum by the evening [43,99,147]. Time of feeding also influences the IRT of external body surfaces due to the heat increment of feeding; temperature starts to increase in cattle approximately 1–2 h after feeding [128,148].

Sunlight, compared to shade, and excessive ambient temperature has positive effects, whilst wind has negative effects on the mean IRT of the eye [91]. Therefore, a thermogram should be taken in a draught free area, where there is low light and the ambient temperature is below 30 °C [138]. A similar distance from lens to subject should be also maintained, to decrease environmental confounding [133]. Turner [138] and Montanholi et al. [128] further recommend repeated scans of an animal until the best quality thermogram is achieved in terms of focus and precise location. However, just one thermogram of an external body surface in a session of thermography may suffice to detect disease [115]. For example, it is possible to detect digital dermatitis in dairy cattle from the maximum IRT of coronary band from only one thermogram/per day [149].

IRT studies show discrepancies in the selection of sample size, thermography session, and thermogram(s) within a session to detect biologically important differences in the IRT of key external body surfaces of cow groups in response to difference in the treatment [134,150,151]. We studied sampling strategy for infrared temperature of lactating dairy cows and reported that the number of replicates thermograms within a session had no effect on the precision of IRT measurements, while the number of thermography sessions, usually taken on different days, had a major effect [32]. To detect meaningful differences in the IRT of left or right or both eyes and coronary band of forelimbs of two groups of cows we recommended to capture two thermograms in quick succession over two sessions on at least 14–16 cows or three sessions on at least 10–12 cows.

We reviewed the various methods of non-invasively measuring heat stress in cattle, highlighting the value of infrared thermography to determine cattle external body surface temperatures. The complexities and usefulness for application of respiratory, body condition, health status, and endocrinological indicators of heat stress have already been summarised at the end of relevant sections. Similarly, the application of infrared thermography is a tricky technique to be applied in field conditions because of some confounding factors as detailed above. The confounding factors can be overcome by installation of thermal cameras in areas with minimum air velocity and no direct exposure of sunlight [131]. The installation of automated thermal cameras with an ability to localise target organs e.g., eyes can be used to measure infrared temperature of cattle in field conditions [152].

## 7. Conclusions

Cattle adopt physiological responses as a coping strategy to heat stress conditions, in particular changes in respiration rate, core body temperature, external body surface infrared temperature, and endocrine hormones. The respiration rate, panting response, and rectal temperature are the most important indicators of heat stress in cattle. The estimation of faecal cortisol metabolites and changes in the infrared temperatures of cattle external body surfaces could also serve as a reliable non-invasive tool for assessing cattle responses to heat stress. IRT measurement offers the best potential to rapidly and non-invasively evaluate heat stress in cattle, but it needs further research to determine optimum methodology.

## Data Availability

Not applicable.
